# The Regulatory Role of Non-Coding RNAs in Autophagy-Dependent Ischemia–Reperfusion Injury of the Brain

**DOI:** 10.3390/cimb47060462

**Published:** 2025-06-17

**Authors:** Irina O. Zakharova, Liubov V. Bayunova, Natalia F. Avrova

**Affiliations:** Sechenov Institute of Evolutionary Physiology and Biochemistry, Russian Academy of Sciences, Thorez av. 44, 194223 St. Petersburg, Russia; zakhar@iephb.ru (I.O.Z.); bayunoval@mail.ru (L.V.B.)

**Keywords:** brain ischemia–reperfusion injury, long non-coding RNAs, microRNAs, circular RNAs, autophagy-related death, protection through autophagy

## Abstract

In recent years, it has become clear that non-coding RNAs play an important role in regulating the development of various organs and pathological conditions, including cerebral ischemia and reperfusion. Non-coding RNAs are mainly represented by long non-coding RNAs (lncRNAs), microRNAs (miRNAs), and circular RNAs (circRNAs). Most of the human genome is transcribed into such RNAs. Excessive activation of autophagy during cerebral ischemia and reperfusion results in autophagic neuronal death in addition to apoptotic death. This review shows that regulation occurs via the lncRNA (or circRNA)/miRNA/target protein signaling axes. A knockdown or a decrease in lncRNA level can lead to a significant increase in miRNA levels, followed by a decrease in the levels of messenger RNA (mRNA) of autophagy-related protein (ATG) and ATG protein itself. This leads to inhibition of autophagy and alleviation of brain ischemia–reperfusion injury. Changes in miRNA and mRNA levels of the target protein occur due to the presence of complementary nucleotide sequences with lncRNA and miRNA, respectively. If the target protein is not an ATG protein, neuroprotection during cerebral ischemia and reperfusion can result from both inhibition and activation of autophagy. The further study of the regulatory role of non-coding RNAs is important as it may help to counteract the effects of excessive autophagy activation and other adverse effects of ischemia–reperfusion injury

## 1. Introduction

The human lifestyle changes including adoption of more sedentary behaviors, shifts in dietary patterns, increased stress levels, and the rising prevalence of obesity and metabolic disorders in different countries could all contribute to the observed growth in health problems. The Global Burden of Disease Study (GBD) is a comprehensive regional and global research program of disease burden that assesses mortality and disability from major diseases, injuries, and risk factors. According to GBD reports, brain ischemic injury or stroke is the second leading cause of death worldwide and a significant contributor to disability [1,2]. The global incidence of ischemic stroke reached 69,944,884.8 cases in 2021, marking a 101.8% increase from 1990 [3]. As the population grows and ages, the number of individuals at risk of brain ischemic stroke increases, leading to a higher absolute burden of the disease. Different projections for the period between 2020 and 2030 predicted that the rate of ischemic stroke would further globally enhance especially in countries with low income [4].

Brain ischemic injury or stroke can be triggered by either vascular occlusion or sudden rupture of cerebral vessels; this is also referred to as ischemic stroke and hemorrhagic stroke, respectively [5]. The prevalence of ischemic stroke is significantly higher than that of hemorrhagic one, accounting for roughly 80% of the total incidence of cerebrovascular injury. In addition, various classifications are used based on different factors, including the duration, severity, and location of ischemia.

According to a systematic review, three main causes of ischemic strokes are known: 50% are caused by arteriosclerotic plaques of the cerebral vessels and the rupture of the arteriosclerotic plaque, 20% are caused by cardiogenic cerebral infarction, and 25% are caused by Lacunar infarcts from small vessel lesions. Furthermore, the remaining 5% are due to other exceptional cause such as vasculitis and extracranial arterial dissection [6]. Ischemic stroke is a heterogeneous condition influenced by a combination of both environmental, lifestyle-related risk and genetic factors [7,8]. The interruption of blood supply to brain, accompanied by hypoxia and nutrient deprivation, further initiates a complex sequence of events that ultimately may culminate in cellular death [9]. Certain populations of nerve cells, for example, CA1 and CA3 pyramidal neurons, are highly susceptible to ischemia. Such vulnerability is based on differences in expressed genome, proteome, metabolome, and transcriptome [10,11].

A reduction in the number of cerebral infarcts, as well as the death of brain tissue and focal damage to neurons after a stroke can be achieved through early restoration of blood flow. Recent therapeutic advances, such as neuroendovascular intervention and thrombolytic therapy, have allowed recanalization of occluded brain arteries in an increasing number of stroke patients [12,13,14]. Early reperfusion leads to positive results even after severe ischemia, which has been demonstrated in the MCAO animal model [15]. Although reperfusion of an ischemic brain is certainly desirable, tissue damage is often the result of both an ischemic stroke itself and the reperfusion process; the latter process causes oxidative stress and an inflammatory reaction that leads to additional damage to the cerebral microcirculation and adjacent brain tissues [16,17,18,19].

The pathophysiology of stroke is rather complicated and involves numerous processes such as energy failure, impaired ion homeostasis, acidosis, excitotoxicity, calcium overload, oxidative stress, disruption of the blood–brain barrier (BBB), mitochondrial dysfunction and neuroinflammation [20,21,22]. All these processes are closely interrelated and lead to profound changes in the activity of neuronal cells and the structure of tissues. As a result, multiple types of cell death have been described in brain ischemia/reperfusion injury [9]. To clarify the situation, the Nomenclature Committee on Cell Death (NCCD) has formulated guidelines for the definition and interpretation of cell death from a morphological, biochemical, and functional point of view. The main types of regulated cell death (RCD) include intrinsic apoptosis, extrinsic apoptosis, mitochondrial permeability transition (MPT)-driven necrosis, necroptosis, ferroptosis, pyroptosis, parthanatos, entotic cell death, NETotic cell death, lysosome-dependent cell death, autophagy-dependent and immunogenic cell death [23].

As far as the effects of severe ischemia and reperfusion are concerned, apoptosis and autophagy seem to be the main reasons for brain injury and neuronal death. Thanks to a wide range of autophagy regulators developed by biotechnology companies, it has become possible to investigate the role of autophagy and autophagic cell death in brain ischemia/reperfusion injuries [24]. The results obtained were controversial. On the one hand, markers of autophagy often increase after excitatory neuronal injury [25] and after the effect of pro-oxidants [26], leading to autophagic neuronal death in addition to apoptotic death of nerve cells. On the other hand, there are cases when activation of autophagy has the protective effect and prevents brain injury. The period when autophagy was considered exclusively as a process protecting cells from death in stress conditions finished many years ago. Now, autophagy is compared with a two-edged sword or the two-faced Janus [27,28]. It seems that if ischemia is severe enough and followed by reperfusion, its activation leads to the autophagic neuronal death.

Such cases are described in the literature. It was shown that a wide range of natural compounds, such as flavonoids, resveratrol, GM1 gangliosides, N-acetylserotonin tetrahydropiperine, catalpol, insulin, substances used in traditional Chinese medicine, as well as synthesized antioxidant X5, were shown to increase the survival of brain neurons by reducing autophagic cell death during ischemic and reperfusion injury [26,29,30,31,32,33,34,35,36,37].

Tetrahydropiperine (THP), a natural arylpentanamide compound isolated from *Piper nigrum* L., demonstrated robust docking capability with proteins associated with the autophagy and PI3K/Akt/mTOR, as indicated by the molecular docking outcomes [36]. The THP significantly reduced behavioral damage by decreasing the area of cerebral infarction and lowering the levels of Atg7 and Beclin-1 proteins. The results of transmission electron microscopy showed no autophagosomes in the THP, 3-methyladenine (3-MA) and 3-MA+THP groups, in addition to the changes in biochemical protein expression of autophagic markers.

The neuroprotective effects of intranasal insulin administration on neurons in CA1 region of the hippocampus and in the brain cortex were completely consistent with the neuroprotective effect of intracerebroventricular injection of 3-MA and apoptotic inhibitor Ac-DEVD-CHO in rats with global brain ischemia caused by two-vessel occlusions [37]. This study demonstrated for the first time the ability of insulin to decrease the autophagic neuronal death, caused by brain ischemia and reperfusion. Insulin administered intranasally activated the Akt kinase (activating the mTORC1 complex, which inhibits autophagy) and inhibited the AMP-activated protein kinase (which activates autophagy) in the hippocampus and frontal cortex of rats with brain ischemia and reperfusion. In this study, the role of autophagy-related death was greater than that of apoptosis-related death.

A list of compounds targeting autophagy is still being updated. Catalpol (CAT), isolated from *Rehmannia glutinosa*, has anti-oxidative and anti-infammatory effects. It has a protective role in the ischemic and reperfused brain. CAT reduced the neurological function deficit and infarct volume and inhibited neuronal apoptotic and autophagic death in the cerebral cortex of MCAO rats [35].

However, in some cases, activation of autophagy in the brain during ischemia and reperfusion can also be protective [38,39,40,41,42]. Autophagy is a conserved and regulated process that occurs within cells, where worn-out proteins and organelles are broken down by lysosomes. This process allows cells to remove damaged components and prevents cell death. Additionally, autophagy serves as an adaptive response, providing cells with nutrients and energy during stressful times, and helps maintain the balance of healthy cells.

Research over the past 15–20 years has led to the discovery of new players involved in the development and the regulation of activity of various organs including brain. Long non-coding RNAs (lncRNAs), circular RNAs (circRNAs), and micro RNA (miRNA) are the types of non-coding RNA that are highly expressed in the central nervous system. For many years the conventional view of gene regulation in biology was centered around protein-coding genes. It was considered that the most important sequence for the development of various organisms was DNA → messenger RNA → protein. However, over the past 15–20 years, it has become clear that the evolution of the developmental processes from primitive organisms to higher vertebrates regulating the complexity of the organism was mainly due to the evolution and expansion of the regulatory potential of the non-coding protein synthesis portions of the genome. Genes encoding non-coding RNA dominate in the genome, and genes encoding protein synthesis make up no more than 2–3% of the genome. In the last 15–20 years, new information was obtained demonstrating the importance of various non-coding RNAs in the regulation of such biological processes as development, differentiation, and metabolism. It gives hope that the new knowledge may be used in future in the therapy of various diseases and pathological states. At the same time, despite the rapid progress of our knowledge about the function of various non-coding RNAs, the function and molecular mechanism of action of the most part of them were not studied yet. Among the numerous publications in which such ideas are developed, the following articles can be noted [43,44,45,46]. The general characteristics of the main type of non-coding RNAs will be given in the next part of the review. The aim of the present review is to characterize the recently obtained data on the regulatory role of non-coding RNAs in autophagy-dependent ischemia–reperfusion injury of brain.

## 2. General Characteristics of Long Non-Coding RNAs, MicroRNAs and Circular RNAs

Recently interesting data were obtained on the role of non-coding RNAs in the regulation of brain metabolism and function and the viability of neurons under various pathological conditions including cerebral ischemia and subsequent reperfusion. It seems strange now that for decades research was focused on the 2–3% of the human genome that encodes protein synthesis. Over the past 15–20 years, it has been discovered that approximately 70–75% of the human genome is transcribed into non-coding RNAs [47,48,49]. It is vital for the regulation of a wide range of biological processes such as growth, development and specific organ function. In addition, non-coding RNAs have been found to play an important role in the regulation of diseases associated with damage and dysfunction of various organs, including the brain [50,51,52,53,54,55,56,57,58]. Non-coding RNAs are currently considered promising targets for drug development.

Non-coding RNAs are mainly represented by long non-coding RNAs (lncRNAs), microRNAs (miRNAs) and circular RNAs (circRNAs). LncRNAs belong to a unique class of RNAs greater than 200 nucleotides in length that have no obvious protein coding functions. There are thousands of lncRNAs in the human and animal body. A comprehensive analysis of lncRNA expression in various organs and regions of the human brain showed that the brain contains much more tissue-specific lncRNAs than other organs [59]. LncRNAs play a post-transcriptional regulatory role; they are able to suppress miRNAs, thereby increasing the production of target proteins.

CircRNAs, characterized by their covalently closed-loop structures without 5′ caps and 3′ poly tails, comprise a large class of non-coding RNAs that are produced by a noncanonical splicing event called back-splicing [60,61]. CircRNAs, unlike traditional linear RNAs, have a closed structure that is not affected by RNA exonucleases [62,63]. For several decades after the discovery of circRNAs, they were considered an operational error, a byproduct of incorrect splicing. Therefore, they have not received adequate attention for a long time [62]. When assessing the impact of various factors, the reliability of circRNAs appears to be important due to different types of false positives [63]. CircRNAs act through specific circRNA–miRNA interactions.

miRNAs belong to a family of non-coding RNAs consisting of 21–25 nucleotides. They have long been the most frequently studied class of non-coding RNAs. They play an important regulatory role in protein synthesis by binding specific target messenger RNAs (mRNAs). By binding to target mRNAs through base pairing, miRNAs negatively regulate mRNA expression via cleavage of mRNA, translation repression or destabilization of mRNA structure, it leads to the decrease in target protein level [49,64].

LncRNA/circRNAs are competing endogenous RNAs (ceRNAs), and they sponge specific miRNAs. Such binding to miRNAs having complementary nucleotide sequences leads to a decrease in the level and activity of the specific miRNA. It reduces the ability of this miRNA to interact with the mRNA of the target protein, which also has complementary nucleotide sequences with the miRNA, and leads to an increase in the level and activity of that target protein. Thus, lncRNAs and circRNAs are capable of indirectly regulating the expression of many genes.

LncRNAs and circRNAs are associated with a wide range of cellular functions. In addition to interacting with microRNAs that have complementary nucleotide sequences, the functional activity of lncRNAs and circRNAs largely depends on their interaction with RNA-binding proteins (RBPs) [65,66]. RBPs interact with coding and non-coding RNAs such as lncRNA and circRNA. RBPs are a large family of proteins that are known to play a critical role in gene expression by modulating RNA splicing, nuclear export, mRNA stability and translation [65,66,67,68]. The effect of non-coding RNAs may depend on their interaction with one or more RBPs. LncRNAs can target many types of proteins; thus, their interaction with transcription factors and proteins of chromatin modification complexes was shown, as well as participation in the post-translational modifications of RBP [66,67]. It was found that in certain cases lncRNAs and circRNAs acted as scaffolds, decoys or guides for RBPs in the process of epigenetic regulation. These non-coding RNAs affect the properties of proteins, which regulate genes at the transcriptional and translational levels [44,65,66,67,68].

## 3. Differentially Expressed Non-Coding RNAs and Messenger RNAs in Ischemic and Reperfused Brain

LncRNAs have been proven to be important gene regulators of development and various diseases. LncRNAs can regulate brain injury and repair by altering the expression of associated genes and proteins. In Duan et al. [69], lncRNA and gene expression profiles were tested in brain tissues of rats subjected to middle cerebral artery occlusion followed by reperfusion (MCAO/R) and sham-operated rats to identify a number of differentially expressed compounds using RNA sequencing. In total, more than 24,000 lncRNAs were screened from six samples using RNA sequencing, the majority of which were lncRNAs detected in both MCAO/R mice and sham-operated animals. A total of 134 differentially expressed lncRNAs (fold change > 2 and *p* < 0.05, false discovery rate *p* < 0.05) and 1006 differentially expressed genes (fold change > 2 and *p* < 0.05) were identified in brain of MCAO/R rats. Among differentially expressed lncRNAs, a total of 77 upregulated and 57 downregulated lncRNAs were identified using RNA sequencing and validated using RT-qPCR in an ischemic stroke group induced by MCAO and reperfusion compared with a control group. Gene Ontology (GO) terms and Kyoto Encyclopedia of Genes and Genomes (KEGG) pathways were used to analyze mRNA functions. An lncRNA–mRNA network was constructed. This study revealed novel differentially expressed lncRNAs. In the Abstract and Discussion, the authors focus on a group of eighteen lncRNAs. It was predicted and then confirmed that these lncRNAs were able to regulate the expression of genes of various substances, such as heme oxygenase 1, mitotic checkpoint serine/threonine kinase B, chemokine ligand 2 and DNA topoisomerase IIα. All of these genes are associated with a cellular response to inorganic substances, alkaloids, estradiol, reactive oxygen species and metal ions. They are involved in various metabolic pathways, chemokine signaling pathways, signaling pathways that determine the cell cycle and other pathways. The authors make conclusion that their study identified novel differentially expressed lncRNAs and an lncRNA-mRNA regulatory network that may allow an improved understanding of the molecular mechanism of ischemic stroke induced by MCAO.

Liu et al. [70] analyzed the expression profiles of lncRNAs in the ischemic brain region of mice after a 45 min of MCAO followed by 48 h of reperfusion. It was found that 255 lncRNAs (217 upregulated and 38 downregulated) and 894 mRNA (870 upregulated and 24 downregulated) showed significantly altered expression in ischemic and reperfused brain compared to sham-operated brain of mice (fold change > 2, *p* < 0.05). To characterize the regulatory effects of lncRNAs differentially expressed in stroke, the authors constructed a co-expression network by combining lncRNAs differentially expressed in ischemic and reperfused brains with differentially expressed mRNAs based on their location, distribution and sequence correlation. The results showed that lncRNAs differentially expressed in stroke interact with differentially expressed genes. The co-expression network showed that one lncRNA is associated with one to dozen of mRNAs. GO and KEGG analyses were performed to determine the function of the differentially expressed mRNAs in ischemic and reperfused brain. The Gene Ontology terms were mainly associated with neutrophil chemotaxis, positive regulation of inflammatory response, cell cycle, positive regulation of apoptotic process. In contrast, the downregulated differentially expressed mRNAs included those that regulate ion transmembrane transport, potassium ion transport and protein phosphorylation.

The functional significance of the activity of non-coding RNA in ischemic pathophysiology was already interesting to the scientists many years ago. Thus, in 2009, Dharap et al. [71] profiled miRNAs in rat brain depending on the time of reperfusion after transient middle cerebral artery occlusion. In total, 238 miRNAs were assessed. In total, 8 of these miRNAs were found to show increased and 12 decreased expression in at least four out of five reperfusion time points examined between 3 h and 3 days, compared with sham-operated rats. Bioinformatics analysis indicated a correlation between miRNAs differentially expressed in ischemic and reperfused brain and several mRNAs known to mediate inflammation, transcription, neuroprotection, receptor function, and ionic homeostasis.

The differentially expressed circRNAs in the brain of rats subjected to MCAO and reperfusion were revealed in the work of Duan et al. [72]. The expression profiles of circRNAs in brain tissues were screened by high-throughput sequencing to identify novel differentially expressed circRNAs. In total, 14,694 circRNAs from the brain of six rats were screened; of these, 87 differentially expressed circRNAS showed a significant fold change > 2 (*p* < 0.05). Quantitative real-time (qRT)-PCR was used to confirm circRNA expression data. In addition, GO and KEGG analyses were performed to investigate mRNA function and to understand the function of circRNAs. Finally, a circRNA-miRNA network was established. In this study, the network of circRNAs–miRNAs–target genes, including 13 miRNAs and their target genes was investigated, indicating that changes in circRNA in ischemic and reperfused brain are associated with genes related with brain injury and recovery.

The work of Liu et al. [73] was also devoted to screening circRNA expression patterns following focal cerebral ischemia and reperfusion; the animals used were mice. The aim of this study was to determine the circRNA expression profiles in the ischemic brain after stroke, which was induced by 45 min of MCAO. The circRNA microarray results showed that 1027 circRNAs were differentially expressed 48 h after beginning of reperfusion in ischemic mouse brains compared with the brains of sham-operated animals. Among them, 914 circRNAs were significantly upregulated, and the remaining 113 were significantly downregulated. In this work, an interaction network of circRNA-miRNA-target genes was constructed, indicating that changes in circRNA are associated with genes related with brain injury and recovery. In these two studies [72,73], it was shown that the differentially expressed circRNA are involved in the regulation of brain injury after stroke, in the regulation of signaling pathways, which are able to make choice between injury and recovery, cell death, or survival.

In Duan et al. [74], miRNA sequencing was performed in the brain infarct area of MCAO/R rats to identify differentially expressed miRNAs. A total of 20 miRNAs with increased expression and 17 miRNAs with decreased activity were found in the infarct area. Subsequently, an miRNA–mRNA network was constructed. Using available bioinformatic data (GO and KEGG), analyzes were performed to explore the mRNA functions targeted by the detected differentially expressed miRNAs. The authors intend to continue their research to check some of their suggestions (for example, that miR-211 may regulate cell proliferation and apoptosis via cGMP-PKG signaling pathway) to better understand the molecular mechanism of action on target proteins of the differentially expressed miRNAs.

A systematic review of the literature from 2015 to 2021 on miRNA expression during acute ischemic stroke was recently conducted by Barrera-Vazquez et al. [75]. The aim was to analyze and identify the most common differentially expressed miRNAs and genes for acute ischemic stroke. A set of miRNAs was revealed, which altered the activity of forkhead boxO3 (FOXO3), FOXO4 and EP300 genes. It is interesting that such genes are involved in cell death and brain-derived neurotrophic factor signaling pathways.

The data recently obtained on differentially expressed non-coding RNAs make contribution to better understanding of the molecular mechanism of brain injury as a result of ischemia and reperfusion and open the possibility of new therapeutic approaches to alleviate the damage.

One of the objectives of the present review is to show how the regulation of autophagy intensity by non-coding RNAs may influence the brain injury and viability of neurons and other cells in ischemic and reperfused brain.

## 4. The Regulatory Effect of Long Non-Coding RNA, MicroRNA and Autophagy-Related Proteins on the Intensity of Autophagy and Brain Ischemia and Reperfusion Injury (LncRNA/MiRNA/ATG Protein and MiRNA/ATG Protein Axes)

The metabolic and functional effects of most non-coding RNAs differentially expressed in ischemic and reperfused brains are still largely unknown, despite the great interest in the problem and the regular acquisition of new interesting data. Our goal is to present recent data on the role of various non-coding RNAs in the regulation of autophagy intensity in the brain, which play an important role in ischemia and reperfusion injury or protection of the brain. In this review, we have already provided examples showing that autophagy is a double-edged sword, as both its inhibition and its activation were found to be protective in various models and studies.

**Figure 1:**
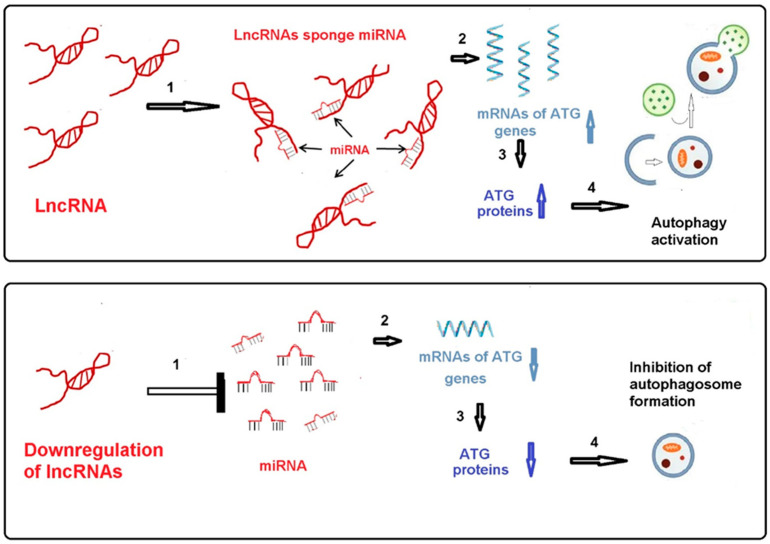
Knockdown of lncRNA leads to a decrease in ATG protein level and inhibition of auto-phagy.

But if the target protein in the lncRNA/miRNA/target protein axis is one of the autophagy-related proteins (ATG protein), then inhibition of cerebral ischemia and reperfusion-activated autophagy appears to be protective in all studies we have encountered in the literature (see Table 1). These studies showed that knockdown of a lncRNA or inhibition of its expression leads to an increase in the level of miRNAs that have complementary nucleotide sequences with this lncRNA. It results in decreased expression of the target ATG protein mRNA (which has complementary nucleotide sequences with the miRNA in its 3′ untranslated region) and decreased levels of that ATG protein. All ATG proteins actively participate in the formation of autophagosomes, thereby activating autophagy. Downregulation of lncRNAs in the lncRNA/miRNA/ATG protein axis indirectly inhibits autophagy and has a protective effect in ischemic and reperfused brain (Figure 1).

To make it clear how this works, let us take a closer look at one of the recent studies. Hu et al. [76] showed that the level of lncRNA PEG11as (lncPEG11as) is significantly increased during transient middle cerebral artery occlusion (MCAO) in mice. To understand the effects of lncPEG11as under such conditions, a lentivirus containing a PEG11as silencing construct (siRNA-PEG11as) was microinjected intracerebroventricularly into males before mice were exposed to MCAO followed by reperfusion (MCAO/R). In MCAO/R mice, knockdown of lncPEG11as expression was shown to prevent excessive activation of autophagy, inhibit neuronal apoptosis, reduce neuronal deficits, and reduce infarct volume. The mechanism of the positive effect of lncPEG11as knockdown was studied. Based on the available bioinformatic data, an miRNA was selected whose interaction had previously been shown with both lncPEG11as and one of the ATG proteins. Among the candidates, miR-874-3p showed high scores. It shares a complementary nucleotide pairing region with both lncPEG11as and the 3′ untranslated region (3′UTR) of mRNA responsible for the synthesis of the ATG16L1 protein. It was then experimentally established [69] that the lncPEG11as/miR-874-3p/ATG16L1 axis is responsible for the protective effect of lncPEG114as knockdown in MCAO/R mice.

The findings suggest that lncPEG11as may be an important mediator involved in the indirect activation of autophagy in the brain under conditions of ischemia and reperfusion. But silencing lncPEG11as alleviates cerebral stroke by inhibiting autophagy and preventing its overactivation in the brains of MCAO/R mice. ATG16L1 is a target protein encoded by the *ATG16L1* gene. This protein is characterized as a subunit of the autophagy-associated complex ATG12/ATG5/ATG16 and is essential for lipidation of LC3-1 (ATG8) (conversion to LC3-II) and autophagosome formation. This complex is localized to the membrane and is released immediately before or after completion of the autophagosome [76]. In this study, various results obtained in vivo were confirmed in vitro using oxygen-glucose deprivation (OGD) of mouse neuroblastoma Neuro 2a cells followed by reperfusion.

In similar studies (see Table 1), other lncRNAs were found to increase in the brain during ischemia and reperfusion and interact with other miRNAs, each of the latter reacting with the mRNA of one of the ATG proteins (ATG3, ATG5b, ATG7, or others) as its target protein [34,77,78,79,80,81,82,83]. In all of these studies, knockdown or downregulation of lncRNAs or upregulation of miRNAs resulted in decreased ATG protein mRNAs and decreased ATG protein levels. In all studies, these changes were shown to be protective by increasing neuronal viability and improving brain function.

## 5. The Regulatory Effect of Long Non-Coding RNAs, MicroRNAs and Various Target Proteins on the Intensity of Autophagy and Brain Ischemia–Reperfusion Injury

Data obtained in the studies of the regulatory effects of non-coding RNAs on the intensity of autophagy and brain ischemia and reperfusion injury show great diversity if the target protein is not an ATG protein (Table 2). In these works, the data of bioinformatic analysis were used to find out the miRNAs, which have complementary amino acid sequences with lncRNA, whose effects were studied. The possible target proteins for the miRNA were also found in bioinformatic data available. But then these data were verified experimentally.

In some studies, alleviation of ischemia and reperfusion injury is achieved by inhibiting autophagy by knocking out or reducing lncRNA levels [84,85,86]. In such studies, lncRNA degrades miRNA and, thus, indirectly increases the amount of mRNA and the level of the target protein. In these three studies, the protective effect was achieved through knockdown or decrease in lncRNA formation or upregulation of miRNA, resulting in inhibition of target protein formation and of excessive activation of autophagy. The same scheme was observed when the target protein was one of the ATG proteins.

Thus, in the work of Deng et al. [85], the target protein was Bcl2/adenovirus E1B 19 kDa interacting protein 3 (BNIP3). This study showed that long non-coding small nucleolar RNA host gene 14 (lncSNHG14) and BNIP3 were highly expressed and the expression level of miR-182-5p was decreased in OGD/R-induced HT22 neuronal cells. LncSNHG14 was found to regulate BNIP3 expression by sponging miR-182-5p. This resulted in excessive activation of autophagy and apoptosis. Knockdown of lncSNHG14 was shown to be protective and prevent death of HT22 cells subjected to OGD and reperfusion.

Li et al. [86] showed that upregulation of miR-202-5p activated the Akt/GSK-3β pathway, reduced infarct volume and ameliorated neurological deficits in rats with transient MCAO. MiR-202-5p was found to accelerate proliferation and suppress autophagy of OGD/R-induced neuronal N2a cells by targeting eukaryotic translation initiation factor 4E (eIF4E). The authors suggest that miR-202-5p may serve as a protective agent against ischemia–reperfusion injury in stroke via eIF4E.

Consistent with these results, a study by Guo et al. [84] showed that long non-coding RNA metastasis-associated lung adenocarcinoma transcript 1 (lncMALAT1), which is a potent autophagy inducer, increases neuronal damage in cortical neurons after OGD and in the mouse cortex after transient MCAO. Suppression of MALAT1 was found to enhance miR-30a expression and attenuate neuronal death through suppression of autophagy in the brain of rats with transient MCAO. To achieve this effect, the MALAT1/miR-30a/Beclin1 axis was used. The autophagy inhibitor 3-methyl-adenine (3-MA) markedly suppressed OGD-induced neuronal death and MCAO-induced ischemic brain infarction. This confirmed the finding that autophagy inhibition plays an important role in the neuroprotective effect of lncMALAT1 silencing.

Interesting data on a different mechanism of autophagy inhibition was reported by Liu et al. [87]. Intracerebroventricular administration of lncAC136007.2 to MCAO rats was shown to reduce the cerebral infarct size and edema in the animals. At the same time, lentivirus-mediated overexpression of AC136007.2 significantly decreased the intensity of autophagic processes in the SH-SY5Y cell line due to inactivation of AMPK/mTOR signaling. Data from Wu et al. [88] also indicate that autophagy inhibition and protective effects can be achieved through lncRNA overexpression. They will be described later.

Thus, a large number of studies showed that excessive activation of autophagy was toxic to neurons under conditions of ischemia and reperfusion, whereas inhibition of autophagy by the regulatory actions of lncRNAs and other non-coding RNAs was protective, even if the target proteins were not ATG proteins [84,85,86,87,88]. But activation of autophagy may also be protective for neurons, as it was shown in other studies [89,90,91,92,93]. Thus, Xue et al. [92] showed that lncRNA taurine activating gene 1 (lncTUG1) was differentially increased in ischemic and reperfused rat brains and in the OGD/R-treated neuronal cell line SH-SY5Y while mitophagy was inhibited. Knockdown of lncTUG1 was shown to promote sirtuin 1 (SIRT1)-induced mitophagy, prevent SIRT1 degradation, and have protective effects. Work by Ren et al. [91] showed that miR-187-3p inhibitor attenuated brain injury induced by ischemia–reperfusion in a rat model of transient MCAO and in OGD/R-treated PC12 cells by restoring seipin expression to improve autophagic flux. Thus, in this study, activation of autophagy had an obvious protective effect.

It seems of interest that the ability of MALAT1 to increase the intensity of autophagic processes may be not only toxic, as it was shown above in the study of Guo et al. [84], but protective as well, as it was shown in brain microvascular endothelial cells (BMEC) [89]. Thus, in BMEC, which play the most important role in blood–brain barrier, a new MALAT1/miR-26b/ULK2 regulatory axis was described. In it, lncMALAT1 served as a competing endogenous RNA by sponging miR-26b and upregulating ULK2 expression, thereby promoting BMEC autophagy and survival under the OGD/R condition [89].

Another study showed that activation of autophagy by non-coding RNAs has a neuroprotective effect. Yao et al. [90] showed that long non-coding small nucleolar RNA host gene 12 (lncSNHG12) expression was increased in the brain of MCAO mice and in neuronal SH-SY5Y cells after OGD and reperfusion. The increase in lncSNHG12 expression was found to alleviate OGD/R-induced damage in SH-SY5Y cells and induce autophagy activation. It has been shown to have protective effects also in vivo in MCAO mice.

But the data obtained in the study of Wu et al. [88] contradict the suggestion that lncSNHG12 can be considered an autophagy inducer [90]. According to Wu et al. [88], the protective effect of lncSNHG12 is due to its ability to inhibit autophagy in HT22 cells. In both studies, activation of lncSNHG12 in a neuronal cell line had a protective effect. These two studies used different neuronal cell lines (SHSY5Y cells and HT22 cells). It can be suggested that in different nerve cells of the brain, lncSNHG12 interacts with different miRNAs. These miRNAs, in turn, interact with various target proteins. This results in either activation or inhibition of autophagy, with protection observed in both cases. But such an assumption requires verification.

Electroacupuncture (EA) treatment has been recommended by the World Health Organization (WHO) for years on cerebral ischemia and reperfusion injury treatment. Large number of investigations are devoted to the studies of specific mechanism of its effect, but it is still far from being fully elucidated. It is clear that EA can relieve brain injury after ischemic stroke by inhibiting various forms of programmed cell death, first of all, such as apoptosis, autophagy and necroptosis. But as far as autophagy is concerned, the protective effect may be achieved both by its inhibition (see, for example [94,95,96]) or its activation (see, for example, ref. [97]). It was shown recently [97] that EA extended the thrombolysis window in rats from 4.5 to 6 h by activation of autophagy, inhibition of apoptosis in endothelial cells and suppression of blood–brain barrier disruption.

Recently, it was shown that non-coding RNAs may be involved in the regulation of the effect of electroacupuncture (EA), its influence on the intensity of autophagy and brain damage in MCAO [93]. It was found that EA of Dazhui, Baihui and Renzhong acupuncture points after MCAO and reperfusion in rats could activate autophagy, improve neurological deficit, and reduce cerebral infarct area ratio and apoptosis rate by promoting the expression of miR-34c-5p, which is beneficial in the treatment of cerebral ischemia and reperfusion injury [93]. Silencing miR-34c resulted in a significantly reduced activating effect of acupuncture on autophagy and increased apoptosis, neurological deficit symptoms, and increased cerebral infarct area ratio. This confirms that EA can upregulate miR-34c-5p expression, which is beneficial in the treatment of cerebral ischemia and reperfusion.

## 6. The Regulatory Effect of Circular RNAs, MicroRNAs and Various Target Proteins on the Intensity of Autophagy and Brain Ischemia–ReperfusionIschemia-Reperfusion Injury

CircRNAs as well as lncRNAs are involved in the regulation of various physiological and pathophysiological processes. Both types of non-coding RNAs are highly expressed in the central nervous system. CircRNAs also take part in the regulation of autophagic processes, cerebral ischemia and reperfusion injury (Table 3).

Jiang et al. [98] showed the elevated expression of circ_0029941 in acute ischemic stroke patients, brain tissue of MCAO/R mice and in OGD/R-induced astrocytes and human glioblastoma cell line A172. It was found that knockdown of circ_0029941 alleviated brain injury, inhibited autophagy induced by ATG5 protein and prevented autophagy and astrocyte activation in MCAO/R mice. In astrocytes and A172 cells knockdown of circ_0029941 also inhibited autophagy and markedly diminished ATG5 and glial fibrillary astrocyte protein (GFAP) level. The authors showed that the axis circ_0029941/miR-224-5p/nuclear factor activated T cell 5 (NFAT5) was responsible for the adverse effects eukaryotic translation initiation factor 4A-III (EIF4A3), which promoted circ_0029941 levels. Circ_0029941 was shown to sponge miR-224-5p and to regulate NFAT5 level. Bioinformatics analysis (Circular RNA Interactome, available at https://circinteractome.nia.nih.gov/index.html accessed on 3 March 2025) showed that there were complementary binding sites between circ_0029941 and miR-224-5p, and their co-localization and interaction were revealed. The bioinformatics analysis (miRDB software, avalible online at https://mirdb.org/, accessed on 3 March 2025) showed that miR-224-5p could bind to the 3′-UTR of NFAT5. NFAT5 mRNA and protein levels could be inhibited by miR-224-5p overexpression and promoted by its inhibition in astrocytes and A172 cells. The data obtained provide evidence that circ_0029941 promotes autophagy to enhance astrocyte activation by miR-224-5p/NFAT5, while knockdown of circ_0029941 leads to alleviation of brain injury.

In the work of Wang et al. [99] the protective effect of circSCMH1, a brain repair-related circular RNA, was studied. In their experiments circSCMH1 was encapsulated into brain-targeting extracellular vesicles mediated by rabies virus glycoprotein. These vesicles were administered to mice with photothrombotic stroke. Such administration of circSCMH1 by brain targeted vesicles enhanced mitochondrial fusion and inhibited mitophagy through suppression of kynurenine 3-monooxygenase (KMO) expression by binding to the transcription activator STAT5B. Thus, circSCMH1 exerted its inhibitory effect on KMO expression, impeding its nuclear translocation. The authors suggest that KMO plays a role in mitochondrial dynamics and mitophagy, independent of its enzymatic function in the kynurenine pathway. KMO accumulation increases the intensity of mitophagy. Overexpression of KMO eliminated the beneficial effect of circSCMH1 administration on behavioral recovery at different time points after stroke as measured by various behavioral tests. KMO is localized in various brain cells. Neurons, astrocytes, microglia, and endothelial cells colocalized with the KMO signal in the peri-infarct cortex. The results obtained provide evidence that circSCMH1 regulates mitophagy through KMO, thereby impacting activation of astrocytes and microglia. The conclusion is made that the novel mechanism by which circSCMH1 downregulates KMO expression was revealed and that such role of circSCMH1 in promoting stroke recovery makes it a therapeutic target for the treatment of ischemic stroke.

It is known that the integrity of the blood–brain barrier (BBB) may be disrupted in various pathological conditions, including cerebral ischemia and reperfusion. Yang et al. [100] found that BBB integrity is damaged by transient MCAO in mice, but BBB damage can be attenuated by circFOXO3, which was shown to activate autophagy through inhibition of mTORC1. Activation of autophagy by circFOXO3 may be suggested to clear cytotoxic aggregates and to improve BBB integrity in mouse brain. In brain microvascular endothelial cells (BMEC) from patients with hemorrhagic transformation, the activation of autophagy was also statistically significant. It was found that OGD/R-induced permeability could be reduced in BMEC if circFOXO3 is overexpressed. Thus, circFOXO3 is able to protect against BBB collapse and appears to be a promising therapeutic target for neurological disorders associated with BBB damage.

Mehta et al. [101] showed that MCAO/R in adult mice decreased the levels of the circular cerebellar degeneration-related protein 1 antisense RNA (circCDR1as) and miRNA-7 (miR-7) levels in the peri-infarct cortex between 3 and 72 h of reperfusion. Decreased miR-7 levels lead to derepression of its major target α-synuclein and secondary brain damage. CircCDR1as is widely distributed in the brain and has more than 70 miR-7 binding sites [102], but its downregulation does not correlate with increased miR-7 levels. Intracerebral injection of an AAV9 vector containing the CDR1as gene significantly increased circCDR1as levels without causing any noticeable toxicity in both the sham and MCAO groups of mice. Overexpression of circCDR1as significantly suppressed α-Syn protein induction after stroke, promoted motor recovery, reduced infarct size, and decreased the levels of markers of autophagy and inflammation in the brain after stroke. The authors suggest that circCDR1as reconstitution is neuroprotective after stroke, probably by protecting miR-7 and preventing α-Syn-mediated neuronal death.

CircHECTD1 levels were increased in ischemic brain tissues during transient MCAO in mice, as well as in plasma samples from acute ischemic stroke patients [102]. CircHECTD1 functions as an endogenous miR-142 sponge; this miRNA is important for normal interactions between neurons and glial cells. Knockdown of circHECTD1 expression increased the level of miR-142 and caused inhibition of autophagy, which led to inhibition of astrocyte activation in mice with transient MCAO, reduction in infarct areas, and attenuation of neurological deficits. This study suggests that circHECTD may serve as a novel biomarker for stroke.

## 7. Conclusions

Over the past 15–20 years, it has been discovered that up to 75% of the human genome is transcribed into non-coding RNAs, while only 2–3% of the genome encodes protein synthesis. Non-coding RNAs play an important role in the regulation of a wide range of biological processes, such as growth, development and specific organ function. In addition, it has been shown that non-coding RNAs are actively involved in the regulation of various diseases and pathological conditions, including ischemia–reperfusion injury of the brain. The non-coding RNAs are represented mainly by lncRNAs, miRNAs and circRNAs. Despite the great interest in the function of non-coding RNAs, and a large number of interesting articles appearing every year, every month and every week, it appears that this is just the beginning of research of the functions of non-coding RNAs. The function of most part of these substances was not studied yet. But perhaps practical success in this area will be achieved before great theoretical achievements. Non-coding RNAs are currently considered promising targets for drug development.

Regulation of autophagy intensity in ischemic and reperfused brain was shown to take place via the signaling axes lncRNA (or circRNA)/miRNA/target protein. In cases of severe cerebral ischemia and reperfusion injury, where excessive activation of autophagy leads to autophagic neuronal death in addition to apoptotic neuronal death, inhibition of autophagy results in pronounced alleviation of brain injury. If the target protein is one of autophagy related (ATG) protein (Table 1), then knockdown of one of the lncRNAs or a significant increase in the level of one of miRNAs was shown to result in a decrease in one of the ATG protein levels due to the decrease in its mRNA level. As ATG proteins actively participate in the formation of autophagosomes, they activate autophagy. So, the decrease in ATG protein level results in autophagy inhibition and decrease in brain damage. Such scheme was valid for all cases that we met in the literature, if the target protein was the ATG protein (Table 1). At the same time, if the target proteins are substances other than the ATG protein, then not only inhibition of autophagy, but also its activation, may be protective (Table 2 and Table 3). Autophagy these days is often compared to a two-faced Janus, although autophagy has long been considered exclusively (or predominantly) a protective process that increases cell viability under stress conditions. We believe that there is a hope that studies of various aspects of regulatory role of non-coding RNAs will offer potential novel strategy for ischemic stroke therapy.

## Figures and Tables

**Table 1:** The regulatory effect of long non-coding RNAs, microRNA and ATG proteins on the intensity of autophagy and brain ischemia and reperfusion injury.

References and the Authors	Models	The Axis or Changes that Regulate Brain Ischemia and Reperfusion Injury	How the Protection Was Achieved	The Changes of Autophagy to Reach the Protection
[34]—Zhang et al., 2023	MCAO/R, rats, PC12 cells (OGD/R)	miR-132-3p/ATG12	MiR-132-3p overexpression	Autophagy was inhibited
[76]—Hu et al., 2022	MCAO/R, mice, Neuro-2a cells (OGD/R)	LncPEG11as/miR-874-3p/ATG16L1	Knockdown of lncPEG11as	Autophagy was inhibited
[77]—Yu et al., 2019	MCAO/R, mice, Neuro2A cells (OGD/R)	LncKCNQ1OT1/miR-200a/FOXO3/ATG7	Knockdown of lncKCNQ1OT1	Autophagy was inhibited
[78]—Yu et al., 2024	Neuro 2a cells (OGD/R)	LncSNHG15/miR-153-3p/ATG5	Downregulation of SNHG15	Autophagy was inhibited
[79]—Cao et al., 2020	MCAO/R, mice, Neuro-2a cells (OGD/R)	LncSNHG3/miR-485/ATG7	Downregulation of lncSNHG3	Autophagy was inhibited
[80]—Wei et al., 2021	MCAO/R, mice, Neuro-2a cells (OGD/R)	LncRMRP/miR-613/ATG3	Knockdown of lncRMRP	Autophagy was inhibited
[81]—Sun et al., 2021	MCAO/R, mice HT22 cells (OGD/R)	LncSNHG14/miR-30b-5p/ATG5	Inhibition of SNHG14 expression by propofol	Autophagy was inhibited
[82]—Wu et al., 2022	MCAO/R, rats	miR-7a/ATG7	Upregulation of miR-7a by sevoflurane	Autophagy was inhibited
[83]—Fan et al., 2023	MCAO/R, rats, cortical neurons (OGD/R)	GATA6/miR-193b/ATG7 (GATA6 is a transcription factor)	Upregulation of miR-193b by GATA6	Autophagy was inhibited

Footnote. MCAO/R—the animals were subjected to middle cerebral artery occlusion followed by reperfusion, OGD/R—the cells of neuronal cell lines or primary neuronal culture were subjected to oxygen and glucose deprivation followed by reperfusion.

**Table 2:** The regulatory effect of long non-coding RNAs, microRNAs and various target proteins on the intensity of autophagy and brain ischemia–reperfusion injury.

References and the Authors	Models	The Axis or Changes that Regulate Brain Ischemia and Reperfusion Injury	How the Protection Was Achieved	The Changes of Autophagy to Reach the Protection
[84]—Guo et al., 2017	MCAO/R, mice, cortical neurons (OGD/R)	LncMalat1/miR-30a/Beclin1	Decrease in lncMALAT1 level	Autophagy was inhibited
[85]—Deng et al., 2020	HT22 cells (OGD/R)	LncSNHG14/miR-182-5p/ BNIP3	MiR-182-5p overexpression	Autophagy was inhibited
[86]—Li et al., 2020	MCAO/R, rats, Neuro-2a cells (OGD/R)	miR-202-5p/elF4E	Upregulation of miR-202-5p	Autophagy was inhibited
[87]—Liu et al., 2021	MCAO/R, rats, SH-SY5Y cells (OGD/R)	Overexpression of Ac136997.2	Overexpression of lncAC136007.2	Autophagy was inhibited
[88]—Wu et al., 2020	HT22 cells (OGD/R)	LncSNHG12 level was increased	Increase in lncSNHG12 level	Autophagy was inhibited
[89]—Li et al., 2017	Primary BMECs (OGD/R)	LncMALAT1/mi-R-26b/ULK2	Decrease in lncMALAT1 level	Autophagy was activated
[90]—Yao et al., 2019	MCAO/R, mice, SH-SY5Y cells (OGD/R)	LncSNHG12 level was increased	Increase in lncSNHG12 level	Autophagy was activated
[91]—Ren et al., 2020	PC12 cells (OGD/R)	MiR-187-3p-Seipin	miR-187-3p inhibition	Autophagy was activated
[92]—Xue et al., 2022	MCAO/R, rats, SH-SY5Y cells (OGD/R)	LncTUG1 is Overexpressed	Knockdown of lncTUG1	Mitotophagy was activated
[93]—Lu et al., 2024	MCAO/R, rats,	Electroacupuncture, miR-34c-5p	Increase in miR-34c-5p expression	Autophagy was activated

Footnote. MCAO/R—the animals were subjected to middle cerebral artery occlusion followed by reperfusion, OGD/R—the cells of neuronal cell lines or primary neuronal culture were subjected to oxygen and glucose deprivation followed by reperfusion.

**Table 3:** The regulatory effect of circular RNAs and microRNAs on the intensity of autophagy and brain ischemia–reperfusionischemia-reperfusion injury.

References and the Authors	Model	The Axis or Changes that Regulate Brain Ischemia and Reperfusion Injury	How the Protection Was Achieved	The Changes of Autophagy to Reach the Protection
[98]—Jiang et al., 2025	MCAO/R, mice, astro- cytes, A172 cells (OGD/R)	EIF4A3/circ_0029941/miR-224-5p/NFAT5	Knockdown of circ_0029941	Autophagy was inhibited
[99]—Wang et al., 2024	MCAO/R, mice	CircSCMH1/KMO	CircSCMH1 suppresses the expression of KMO	Mitophagy was inhibited
[100]—Yang et al., 2022	MCAO/R, mice, BMECs (OGD/R)	CircFOXO3	Upregulation of circFOXO3	Autophagy was activated
[101]—Mehta et al., 2022	MCAO/R, rats	CircCDR1as, miR-7	Overexpression of circCDR1as	Autophagy was inhibited
[102]—Han et al., 2018	MCAO/R, mice	CircHECTD/miR-142	Knock-down of circHECTD	Autophagy was inhibited

Footnote. MCAO/R—the animals were subjected to middle cerebral artery occlusion followed by reperfusion, OGD/R—the cells were subjected to oxygen and glucose deprivation followed by reperfusion, EIF4A3—eukaryotic translation initiation factor 4A-III, NFAT5—nuclear factor activated T cell 5, A172—human astrocytoma cell line, KMO—kynurenine 3-monooxygenase, BMECs—brain microvascular endothelial cells.

## Data Availability

The electronic bibliographic database PubMed was used to search then references.

## Abbreviations

The following abbreviations are used in this manuscript:

AKT	Protein kinase B
AMPK	Adenosine monophosphate-activated protein kinase
ATG proteins	Autophagy-related proteins
BBB	Blood–brain barrier
BMEC	Brain microvascular endothelial cells
BNIP3	Bcl2/adenovirus E1B 19 kDa interacting protein 3
ceRNAs	Competing endogenous RNAs
circCDR1as	Circular cerebellar degeneration-related protein 1 antisense RNA
circRNAs	Circular RNAs
EA	Electroacupuncture
eIF4E	Eukaryotic translation initiation factor 4E
EP300	E1A-associated protein p300 (E1A = adenovirus early region 1A)
FOXO3	Forkhead box O3
GO	Gene Ontology
HT22	Mouse hippocampal neuronal cell line
KEGG	Kyoto Encyclopedia of Genes and Genomes
LC3	Light chain 3
lncKCNQ1OT1	Long non-coding RNA potassium voltage-gated channel subfamily Q member 1opposite strand 1
lncMalat1	Long non-coding RNA metastasis-associated lung adenocarcinoma transcript-1
lncRNAs	Long non-coding RNAs
lncPEG11as	Long non-coding paternally expressed gene 11 antisense transcript
lncSNHG15	Long non-coding RNA small nucleolar RNA host gene 15
lncTUG1	LncRNA taurine activating gene
MAPK	Mitogen-activated protein kinase
MCAO	Middle cerebral artery occlusion
MCAO/R	Middle cerebral artery occlusion and reperfusion
miRNAs	microRNAs
mTOR	The mammalian/mechanistic target of rapamycin
OGD	Oxygen and glucose deprivation
PC12	Pheochromocytoma cell line of the rat adrenal medulla
SH-SY5Y	Stable human neuroblastoma cell line
SIRT1	Sirtuin 1
ULK1/2	Unc-51-like kinase 1/2
